# Global surgery initiatives for collaborative surgical education and research

**DOI:** 10.1016/j.amsu.2021.102583

**Published:** 2021-07-26

**Authors:** Mohammad Aadil Qamar, Irfan Ullah, Abdul Moiz Sahito, Talal Almas, Abdul Waris, M. Ali Kanawati, Mohammad Almuhaileej, Asimina Dominari

**Affiliations:** a Ziauddin Medical University, Karachi, Pakistan; b Kabir Medical College, Gandhara University, Peshawar, Pakistan; c Dow Medical College, Dow University of Health Sciences, Karachi, Pakistan; d RCSI University of Medicine and Health Sciences, Dublin, Ireland; e Aristotle University of Thessaloniki School of Medicine, Thessaloniki, Greece

## Abstract

As technology advances, sharing data instantaneously is becoming easier than ever and opportunities for international collaborations are becoming more and more easily available on a virtual level. Amongst the numerous areas of expertise that could benefit from this development, surgery stands out to be an esoteric one given the challenges faced as one embarks on collaboration in surgical education and research. Herein, we delve into the challenges faced when such international collaborations are attempted and provide insight as to how different areas across the globe could collaborate to improve outcomes in surgical education and research.

With more than 190 countries in the world and the vast variation of standards in surgical education and research, trying to achieve a common goal might be quite difficult. Problems can arise because of the differences among the various countries and/or geographic areas, ranging from the varying availability of resources and expertise, to the different cultures and mindsets with respect to education and research. Moreover, lack of time and engagement can always become an obstacle along the way.

Despite the many potential issues, it is remarkable how low- and middle-income countries (LMICs) work together with their counterparts from high-income countries (HICs), especially in times of crisis. A recent example of global collaboration is the extensive sharing of data during the COVID-19 pandemic. Instantaneous availability of scientific data and healthcare-related information helps open a vast array of opportunities such as research and the consequent developments, which would otherwise not be available in many parts of the world due to various reasons, such as politics, religious inclinations etc. [1].

Funding has always been a major concern especially for researchers from LMICs. The lack of resources in such countries makes it difficult to work towards the publication of high-impact work. A good comparison of LMIC versus HIC spending on Research and Development (R&D) would be to compare the R&D spending as a percentage of the Gross Domestic Product (GDP) in Pakistan and the United States of America (USA). It was found that Pakistan spends a mere 0.3 % ($2298.5 million) of GDP as R&D spending, whereas the USA spends 2.7 % ($476,459 million) [2]. Given such statistics, one realizes the disparity in resources available in different countries. This becomes a major hindrance that requires attention. On the other hand, Arab countries are likely to have lower published medical studies compared to the international average [3]. This is an average of 189 medical studies per million inhabitants [4]. A bibliometric study looking for the published biomedical studies between 2001 and 2005 reported that Arab countries reported fewer publications compared to other middle eastern countries [4]. Moreover, when measuring the quality of the research using impact factor criteria and the H-index, the studies tend to be of lower quality [5]. Moreover, the expenditures for scientific research in the Arab countries is 0.2–0.4% of the national income growth domestic product compared to 4–6% in the developed countries [6].

Taking the example of the observed case of Covid-19 vaccine in which Covax will be ensuring equitable access to vaccines to countries which otherwise do not have the resources to vaccinate its entire population, such an authority ensures maintenance of balance that is required for global collaborative programs including research and education. A global authority that could ensure a fair distribution of funds as well as expertise across the globe with the help of better equipped countries, provided a dedicated budget for the purpose will ensure coordinated research and funding strategies are in place for surgical related initiatives as is seen in other sectors of medical care [7,8].

The Global Paediatric Surgery Network (GPSN) provides a model for understanding how global efforts can be undertaken [9]. Countering issues related to finance, partnerships, and education, this organization helps reduce the barriers. A simple step towards the right direction can be seen in waiving of membership fees. This practice encourages researchers from LMICs to actively participate in the advancement of surgical research and education. The opportunity for researchers from LMICs to contribute to the current literature while simultaneously bringing back the acquired knowledge to their respective countries, will help in the improvement of surgical expertise in their countries. Another novel approach to further the effort of global collaboration is seen in the creation of various databases which act as a reservoir of information available at our fingertips. Such a practice has been used for the measurement of the six surgical metrics recommended in the Lancet Commission on Global Surgery [8,10]. These metrics measure the global surgical indicators across regions which is important given it provides us with the opportunity to compare between similar nations and consequently push for improvements. In addition to the mentioned initiative, creation of consultant groups on the electronic media helps in case discussion and improved management in the field. The added benefit of a two-way promotion of knowledge sharing in case of innovative low-cost ideas seen in LMICs help in improving medical practices in HICs as well. We see such efforts being underway to a lesser extent in global surgery. Organizations such as ‘GlobalSurg and the NIHR Global Health Research Unit on Global Surgery’, ‘InciSioN’ etc. are a step in the right direction [[11], [12], [13]]. The incorporation of young minds in the form of students, trainees, residents etc. with experienced researchers help in the creation of the next generation of researchers who will be equipped to further improve the cause of joint work across the globe. Creation of such organizations is imperative given the outlook of surgical research needs collaboration from every corner of the world to improve outcomes in the quest for better service delivery.

Coming back to the comparison between Pakistan (LMICs) vs the United States of America (HICs), we now look at the number of researchers for each country. The UNESCO (United Nations Education, Scientific and Cultural Organization) Institute for Statistics states that in Pakistan there are 158 researchers for every million inhabitants whereas in the United States of America there are 4205 researchers for every million inhabitants [2]. A glimpse into the mammoth difference in resources again. Bridging of these differences has been seen via various efforts such as inclusion of elective training at an LMIC during training years at a more well-resourced organization, conduction of workshops, conferences, symposia, providing grants to younger doctors from low-income households, incorporation of scholarships/financial grants into training programs etc. [9,[14], [15], [16]]. Such efforts help the young generation in broadening their expertise as they make global networks for collaborations and learn from their peers in HICs [17]. It is however unfortunate when at times, time constraints keep such bright minds held back. This issue has been dealt with to some extent by the formal introduction of designated time during training years where trainees are provided with the time to focus on such activities but then again, this plan is more prevalent in HICs rather than LMICs [18,19].

Looking at the induction of various ways to promote global collaboration in research and education, it can be reliably concluded that establishment of designated funds for supporting researchers from LMICs can go a long way towards improving conditions in which HICs need to play an active role to aid their counterparts. In addition to this, organizations in LMICs need to incorporate activities such as updated training, workshops, local conferences, audits etc. to improve their facilities as well as expertise at an institutional level to play their part in their respective countries. In a time where collaboration is needed more than ever, being left back due to reasons in our control will be a tragedy many might not comprehend until it might be too late.

We recommend the following points:

1. To establish collaborative bridges between the western developed countries with other incapable countries in research and experiences exchange.
2. To help gifted and interested surgeons with training and international research programs to build their knowledge and experience to do the same in their countries.
3. To put international laws and rules for research and data exchanges between research centers in all the world.

During this age of technology, sharing data instantaneously has become easier than ever. Such an opportunity provides with itself the prime chance of excelling in our respective field of expertise together with global collaborative initiatives. Amongst the many specialties of the medical world, surgery stands out to be an esoteric one given the challenges faced as one embarks on collaboration in surgical education and research. There is thus an overarching need to delve into these challenges and provide insight as to how different areas across the globe work in conjunction to improve outcomes in surgical education and research.

## Please state any conflicts of interest

None.

## Please state any sources of funding for your research

None.

## Ethical approval

NA.

## Consent

NA.

IA: Study concept or design.

MAQ, IA, AMS, TA, AW, AD: Data collection, data analysis or interpretation, writing the paper.

MAK, MA, AD: Critical revision of the article.

## Registration of research studies

Name of the registry: NA
Unique Identifying number or registration ID: NA
Hyperlink to your specific registration (must be publicly accessible and will be checked): NA

## Guarantor

Talal Almas RCSI University of Medicine and Health Sciences 123 St. Stephen's Green Dublin 2, Ireland Talalamas.almas@gmail.com +353834212442.
